# Transcriptome analysis reveals genes connected to temperature adaptation in juvenile antarctic krill *Euphausia superba*

**DOI:** 10.1007/s13258-023-01377-7

**Published:** 2023-06-10

**Authors:** Yongliang Liu, Lingzhi Li, Jialiang Yang, Hongliang Huang, Wei Song

**Affiliations:** 1 School of Ocean, Yantai University, 30 Qingquan Road, Yantai, Shangdong, 264005 China; 2 Key Laboratory of Oceanic and Polar Fisheries, Ministry of Agriculture, East China Sea Fisheries Research Institute, Chinese Academy of Fishery Sciences, Shanghai, 200090 China

**Keywords:** *Euphausia superba*, Transcriptome analysis, Differentially expressed genes, Temperature adaptation

## Abstract

**Background:**

The Antarctic krill, *Euphausia superba* (*E. superba*), is a key organism in the Antarctic marine ecosystem and has been widely studied. However, there is a lack of transcriptome data focusing on temperature responses.

**Methods:**

In this study, we performed transcriptome sequencing of *E. superba* samples exposed to three different temperatures: −1.19 °C (low temperature, LT), − 0.37 °C (medium temperature, MT), and 3 °C (high temperature, HT).

**Results:**

Illumina sequencing generated 772,109,224 clean reads from the three temperature groups. In total, 1,623, 142, and 842 genes were differentially expressed in MT versus LT, HT versus LT, and HT versus MT, respectively. Moreover, Kyoto Encyclopedia of Genes and Genomes analysis revealed that these differentially expressed genes were mainly involved in the Hippo signaling pathway, MAPK signaling pathway, and Toll−like receptor signaling pathway. Quantitative reverse-transcription PCR revealed that ESG037073 expression was significantly upregulated in the MT group compared with the LT group, and ESG037998 expression was significantly higher in the HT group than in the LT group.

**Conclusions:**

This is the first transcriptome analysis of *E. superba* exposed to three different temperatures. Our results provide valuable resources for further studies on the molecular mechanisms underlying temperature adaptation in *E*. *superba*.

## Introduction

The Antarctic krill (*Euphausia superba; E. superba*) belongs to the family Euphausiidae (Parker and Tyedmers 2012). It is one of the most abundant biological organisms on earth and plays a key role in maintaining balance in the Antarctic marine ecosystem (Croxall et al. 1999). Studies have shown that the current global biomass of Antarctic krill exceeds 300 million tons (Atkinson et al. 2009). *E. superba* has also been linked to iron recycling and the production and uptake of ammonium (Tovar-Sanchez et al. 2007; Whitehouse et al. 2011). *E. superba* is rich in proteins and many essential nutrients, including phospholipid polyunsaturated fatty acids, chitin, astaxanthin, and enzymes that are active at very low temperatures (Tou et al. 2007). Many companies are engaged in the development of high-value krill products, including Antarctic krill meal and Antarctic krill oil (Backes and Howard 2014; Nicol et al. 2012). Suontama et al. (2010) discovered that Antarctic krill meal could promote the growth of Atlantic salmon and Atlantic halibut. Zhou et al. (2016) found that Antarctic krill oil could lower blood pressure in spontaneously hypertensive rats. Thus, *E. superba* not only plays a crucial role in the Antarctic ecosystem but also serves as an increasingly important resource for commercial fisheries in the Southern Ocean. Therefore, it is necessary to gain insights into the genetic resources of *E. superba* to facilitate such commercial applications while protecting this key organism.

The distribution of *E. superba* has undergone significant changes over the last few years owing to ongoing environmental changes, including rising temperatures, declining sea ice levels, and ocean acidification (Hill et al. 2013). Of these, temperature is one of the most fundamental and important factors underlying the distribution changes. Temperature can affect the survival and growth of *E. superba* (Brown et al. 2010); even small changes, between 1 and 2 °C, can result in significant alterations to the physiological properties, distribution, and behavior of these organisms (Flores et al. 2012; Piñones and Fedorov 2016). Atkinson et al. (2006) reported that *E. superba* cannot tolerate temperatures above 3.5 °C for long periods of time. Therefore, it is crucial to understand the adaptability of *E. superba* to different temperatures.

The rapid development of high-throughput sequencing technology has facilitated the wide-scale adoption of RNA sequencing (RNA-seq) as a tool for transcriptome profiling. An increasing number of transcriptional studies that focus on the molecular response of organisms to various environmental factors are being conducted as researchers try to understand the mechanisms through which such organisms adapt to their changing environments. Li et al. (2017) performed transcriptome analysis of rainbow trout liver under moderate heat stress and discovered numerous heat shock protein genes for temperature regulation and some genes related to heat stress. Shi et al. (2019) found that the endoplasmic reticulum stress response plays a crucial role in the adaptation of Atlantic salmon to heat stress via RNA-seq. Zhou et al. (2019) conducted transcriptome analysis of Nile tilapia under low temperature and uncovered that cold stress can drive kidney disfunction and inhibit the immune-associated pathways in Nile tilapia. However, there is lack of research on transcriptome analysis for temperature adaptation in *E. superba*.

In the present study, we analyze RNA-seq data from *E. superba* exposed to three different temperatures based on the KrillDB2 database. We first determined the differentially expressed genes (DEGs) and pathways during high-temperature stress. Afterward, the expression of genes involved in pathways related to stress was verified by quantitative reverse-transcription PCR (qRT-PCR). We anticipate that our data will form a solid foundation for further research focused on identifying the mechanisms underlying temperature adaptation in *E. superba*.

## Materials and methods

### Animal collection

Juvenile *E. superba* (29.91 ± 2.35 mm length) was collected from the Amundsen Sea (70°57′37″ S, 125°55′08″ W) using a pelagic net (RMT8), based on the observation of krill using an EK60 echo sounder (BioSonics, USA) on March 11, 2018. Healthy live juvenile krills were moved to a flow-through tank on board, which was continuously fed with seawater at -0.5-+0.5  °C. Krills were adapted to aquarium conditions for 24 h before the start of experimentation to minimize holding stress. A circulation pump was employed to set up a vertical current to keep the krills in the water column.

The same aquarium system was used for different temperature treatment. The krill were exposed to three culture conditions: a low temperature group (− 1.19 °C, LT), a medium temperature group (− 0.37 °C, MT), and a high temperature group (3 °C, HT) for 48 h. These temperature conditions were maintained using a temperature-programmable incubator (Thermo Precision Model 818, MA, USA). Notably, −1.19  °C was the temperature of the water in which *E. superba* were collected, − 0.37 °C was the ambient air temperature, and 3 °C was the higher temperature that *E. superba* are known to tolerate (Atkinson et al. 2006). We exposed *E. superba* to the three different temperatures for 48 h. Each sample included three replicates. After 48 h of temperature treatment, the krill samples of each group were independently collected and saved in RNAlater (TransGen Biotech, Beijing, China) and then stored at − 80 °C prior to RNA extraction. All sample collection and animal handling procedures in this study were approved by the Ethics Committee of Chinese Academy of Fishery Sciences.

### RNA extraction, complementary DNA (cDNA) library preparation, and sequencing

Total RNA from the whole body of each sample was extracted using TRIzol reagent (Invitrogen, Carlsbad, CA, USA) according to the manufacturer’s protocol. Total RNA concentration was measured using a Nanodrop 2000 (Invitrogen, Carlsbad, CA, USA), and RNA integrity was evaluated using an Agilent 2100 Bioanalyzer (Agilent Technologies, CA, USA). Samples were deemed to be of sufficient quality when the RNA integrity number was between 7 and 10. Each library was constructed of no less than 5 µg of RNA. Qualified samples were stored in an ultra-low temperature freezer at − 80 °C. cDNA libraries were constructed using the Illumina TruSeq™ RNA Sample Preparation Kit (Illumina, San Diego, USA). Briefly, mRNA was isolated using magnetic beads conjugated to oligo. Fragmentation was performed in fragmentation buffer. Using mRNA as a template, the first cDNA strand was synthesized using random primers. Second-strand cDNA was synthesized using DNA polymerase I and ribonuclease H. Then, the adapter was ligated after adenylation of the 3ʹ ends of the DNA fragments. The target fragments were recovered using 2% agarose gel electrophoresis, and PCR amplification was performed to construct the sequencing library. The resultant cDNA libraries were sequenced on an Illumina HiSeq 2000 platform (Illumina, USA).

### Processing of raw sequences and analysis

Raw reads were qualified using Fast-QC software (v0.11.4). To obtain high-quality data for analysis, raw reads were cleaned by removing reads with adapters, those with “N” (representing an ambiguous base), and more than 20% bases with *Q* < 20. The entire transcript sequences of *E. superba* were downloaded from the KrillDB2 database (https://krilldb2.bio.unipd.it/). Our data was mapped to the database for the subsequent analysis.

### DEGs identification and functional enrichment analysis

Clean reads of each sample were matched to KrillDB2 using salmon software (version 1.9.0). We used tximport package (version 1.24.0) of R software (version 4.2.1) to import data of RNA-seq, and then used DESeq2 (version 1.36.0) software to identify DEGs. The threshold parameter for significant differential expression was set at FDR < 0.05 and |log_2_ (fold change)| > 1.

We downloaded the gene ontology (GO) annotation information from the KrillDB2 database. We used online tools KAAS (https://www.genome.jp/tools/kaas/) to calculate homologous relationship between the Antarctic krill genes and Kyoto Encyclopedia of Genes and Genomes (KEGG) genes. GO and KEGG enrichment analysis was performed using the R software package clusterProfiler (version 4.4.4).

### qRT-PCR

To verify the RNA-seq results, four DEGs with large expression changes involved in temperature adaptation-related pathways were chosen for qRT-PCR analysis using the same RNA samples used in the Illumina sequencing. Gene-specific primers were designed using Primer Premier 5.0. Ribosomal protein S13 (RPS13) was used as an internal reference gene. cDNA was synthesized using the RevertAid First Strand cDNA Synthesis Kit (Thermo Fisher Scientific, MA, USA). qRT-PCR was performed using an ABI Q6 real-time PCR machine (Applied Biosystems, Foster City, CA, USA). The reaction mixture (10 µL) contained 5 µL 2 × Master Mix, 0.3 µL of each primer, 1 µL cDNA, and 3.4 µL ddH_2_O. The PCR cycling parameters were as follows: 95 °C for 10 min, 45 cycles of 95 °C for 15 s and 60 °C for 60 s, followed by a melt curve. Each sample was analyzed in triplicate, and the amplicons were verified by melt-curve analysis. The 2^−ΔΔCT^ method was used to determine the relative gene expression for each DEG, and the quantitative data are expressed as the mean ± standard deviation (SD; n = 3). Significant differences in gene expression were determined using a *t*-test with a significant level of *p* < 0.05.

## Results

### Overview of transcriptome sequencing

Using the Illumina HiSeq 2000 platform, transcriptome sequencing was performed in the LT, MT, and HT groups. The results of the analysis of the clean reads were shown in Table 1. After removing the low quality reads, 273,920,342, 227,339,348, and 270,849,534 clean reads were generated from the LT, MT, and HT groups, respectively. In total, we obtained 772,109,224 clean reads. The base mass-value ratio of Q30 in each sample was not less than 86.3%, and the GC content was between 38.52% and 41.84%. These results indicated that the transcriptome sequencing data were of high quantity and quality, which could provide reliable original data for subsequent analysis.

**Table 1:** Analysis of the clean reads from *E. superba* exposed to different temperatures (LT, MT, and HT). LT: low temperature (− 1.19 °C); MT: medium temperature (− 0.37 °C); HT: high temperature (3 °C)

Sample	Number of sequences	Number of bases	Q (20%)	Q (30%)	GC (%)
LT-1	87,947,502	13,091,812,701	97.47	93.75	39.97
LT-2	89,574,906	13,261,971,873	93.93	86.35	39.81
LT-3	96,397,934	14,361,213,996	98.15	95.53	40.55
MT-1	92,041,470	13,719,124,215	97.41	93.59	40.18
MT-2	74,093,036	10,957,807,196	93.92	86.3	41.84
MT-3	61,204,842	9,108,055,218	98.18	95.63	41.6
HT-1	95,192,326	14,191,897,080	97.54	93.89	38.52
HT-2	87,639,746	12,972,156,832	93.92	86.31	40.93
HT-3 Total	88,017,462 772,109,224	13,105,456,393 114,769,495,504	98.1 -	95.47 -	40.61 -

### Analysis of DEGs

**Fig. 1:**
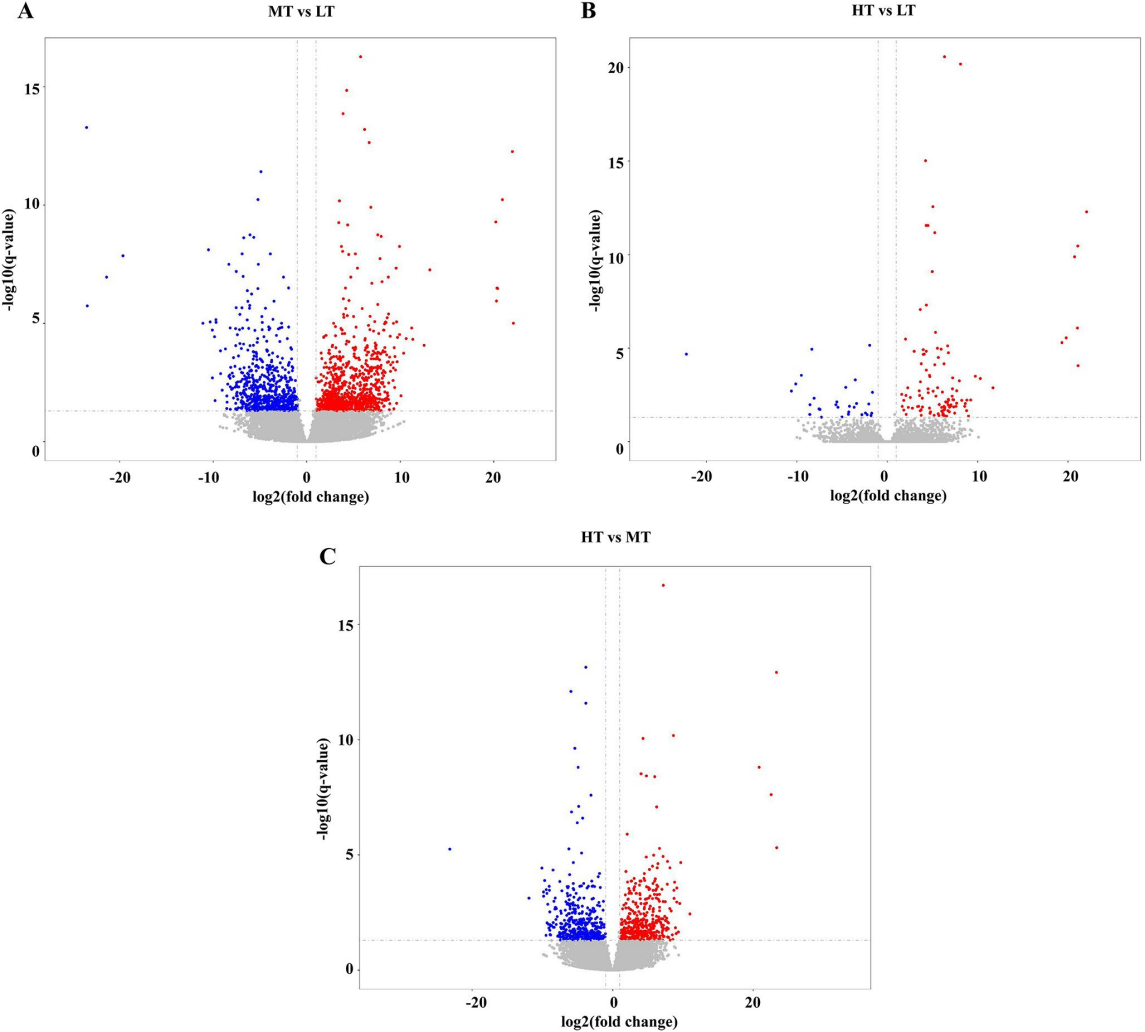
Distribution analysis of differentially expressed genes (DEGs). (A) DEGs in MT vs. LT. (B) DEGs in HT vs. LT. (C) DEGs in HT vs. MT. Red dots represent significantly upregulated genes. Blue dots represent significantly downregulated genes. Gray dots represent non-significant DEGs. Each dot represents one gene. LT: low temperature (− 1.19 °C); MT: medium temperature (− 0.37 °C); HT: high temperature (3 °C)

To search for genes associated with temperature adaptation, we identified DEGs from *E. superba* under different temperatures. A total of 1,623 DEGs were identified between the MT and LT groups, of which 930 DEGs were upregulated and 693 DEGs were downregulated in the MT group (Fig. 1A). In addition, 142 DEGs were identified between the HT and LT groups, of which 112 were upregulated and 30 were downregulated in the HT group (Fig. 1B). Moreover, 842 DEGs were identified between the HT and MT groups, of which 416 were upregulated and 426 were downregulated in the HT group (Fig. 1C).

### GO enrichment analysis of DEGs

**Fig. 2:**
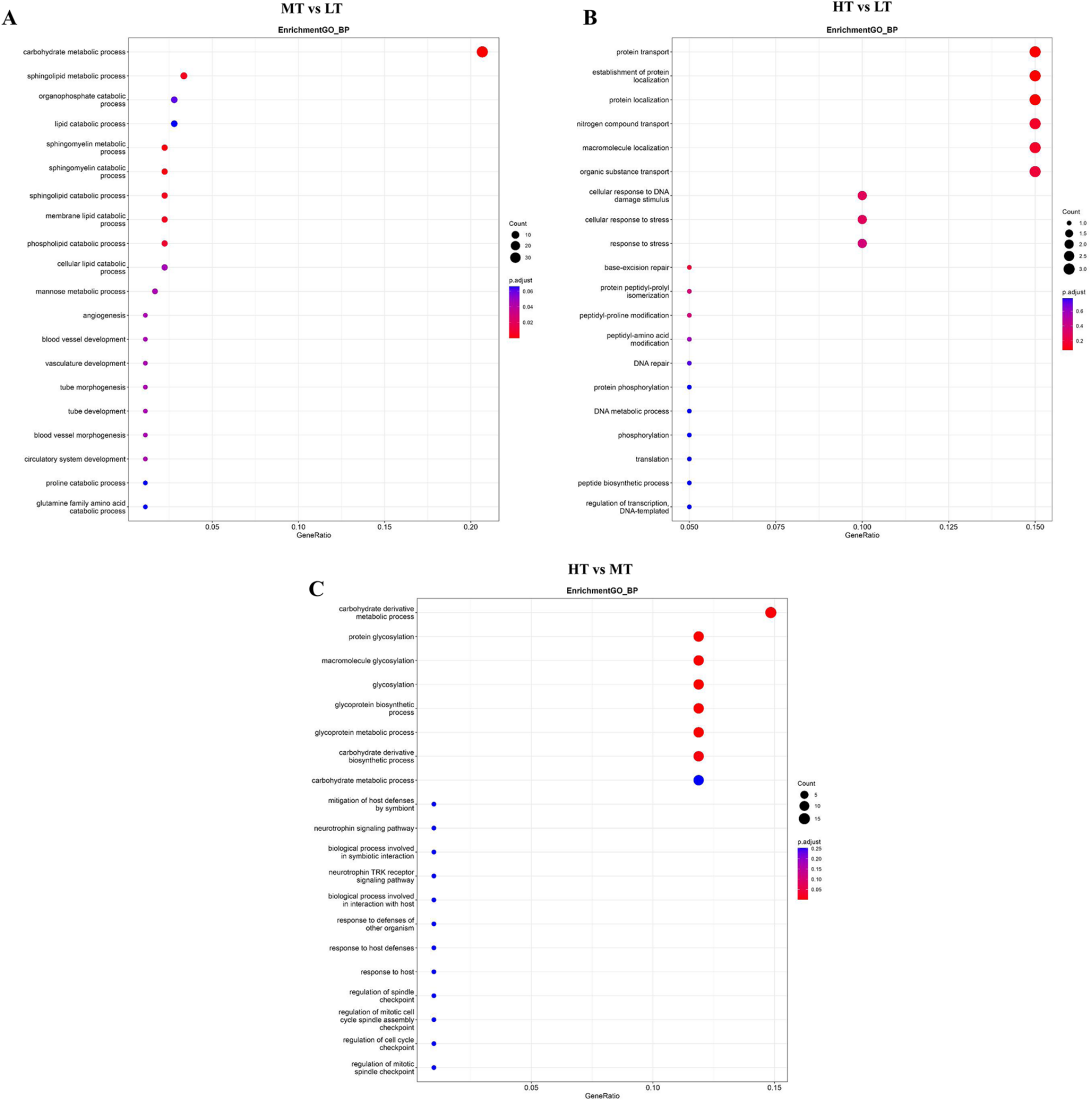
Gene Ontology (GO) enrichment analysis of differentially expressed genes (DEGs) from *E. superba* based on biological processes. (A) GO analysis of DEGs in MT vs. LT. (B) GO analysis of DEGs in HT vs. LT. (C) GO analysis of DEGs in HT vs. MT. The size of the circle in the figure represents the number of DEGs. The color represents the significance of the p value

To understand the functions of the DEGs, we performed GO analysis based on biological processes (BP) categories. The BP categories analysis showed that DEGs between MT and LT were mainly involved in carbohydrate metabolic process, sphingomyelin metabolic process, and lipid catabolic process (Fig. 2A). DEGs between HT and LT were mainly involved in protein transport, establishment of protein localization, protein localization, and cellular response to stress (Fig. 2B). DEGs between HT and MT were mainly involved in protein glycosylation, macromolecule glycosylation, and glycosylation (Fig. 2C). Therefore, these results indicated that DEGs were primarily enriched in GO terms related to glucose metabolism, protein processing and cellular activities, suggesting that it may be related to temperature regulation.

### KEGG enrichment analysis of DEGs

**Fig. 3:**
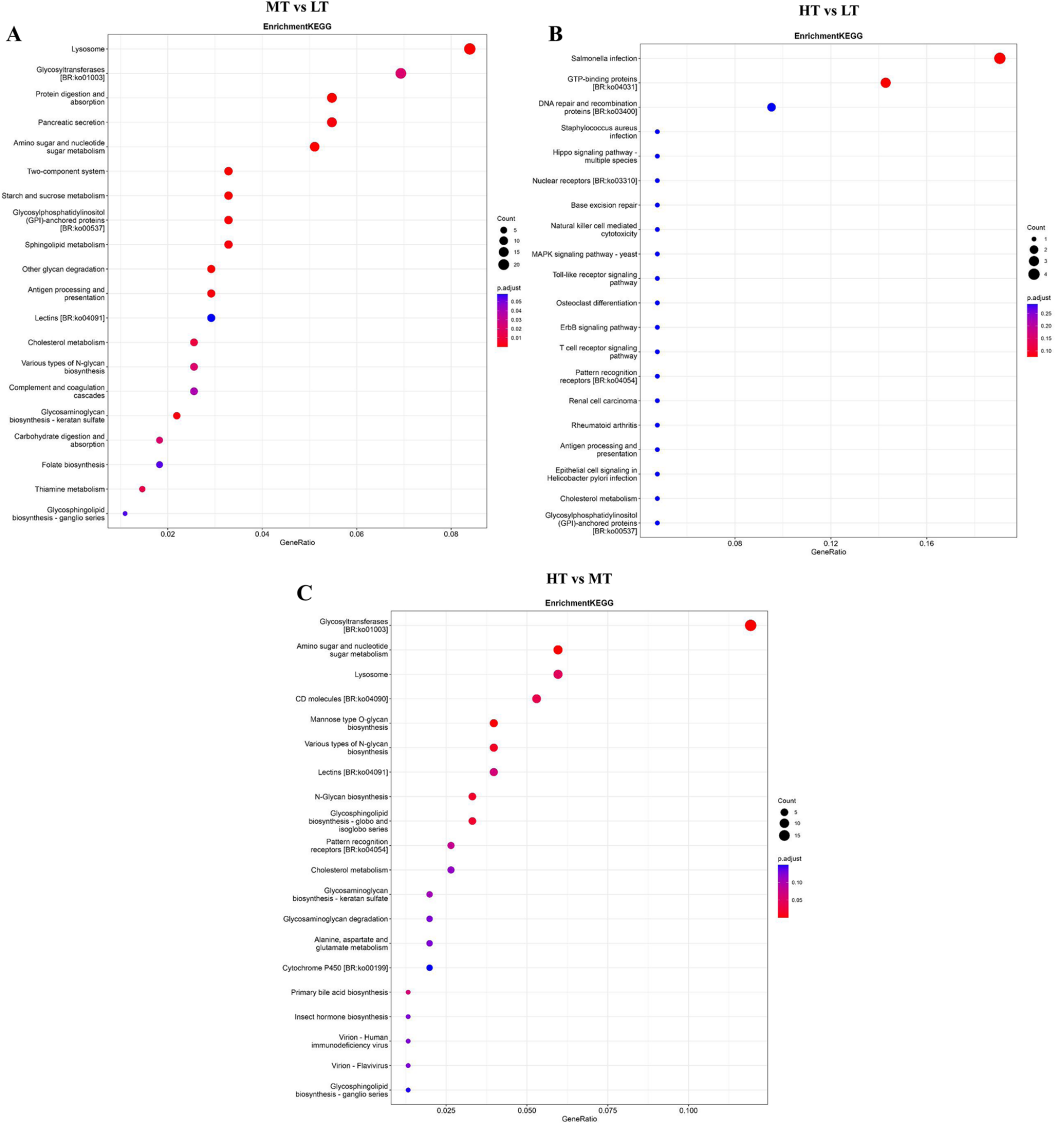
Kyoto Encyclopedia of Genes and Genomes (KEGG) enrichment analysis of the differentially expressed genes (DEGs). (A) KEGG analysis of DEGs in MT vs. LT. (B) KEGG analysis of DEGs in HT vs. LT. (C) KEGG analysis of DEGs in HT vs. MT. The size of the circle in the figure represents the number of DEGs. The color represents the significance of the p value

To understand the pathway of the DEGs, we performed KEGG enrichment analysis. KEGG analysis revealed that DEGs between MT and LT were mainly involved in protein digestion and absorption, amino sugar and nucleotide sugar metabolism, and starch and sucrose metabolism (Fig. 3A). DEGs between HT and LT principally participated in Hippo signaling pathway, MAPK signaling pathway, and Toll−like receptor signaling pathway (Fig. 3B). DEGs between HT and MT were primarily involved in amino sugar and nucleotide sugar metabolism, mannose type O−glycan biosynthesis, and N−Glycan biosynthesis (Fig. 3C). These results indicated that DEGs might participate in temperature adaptation by affecting the activation of the above pathways.

### Validation of gene expression using qRT-PCR

**Fig. 4:**
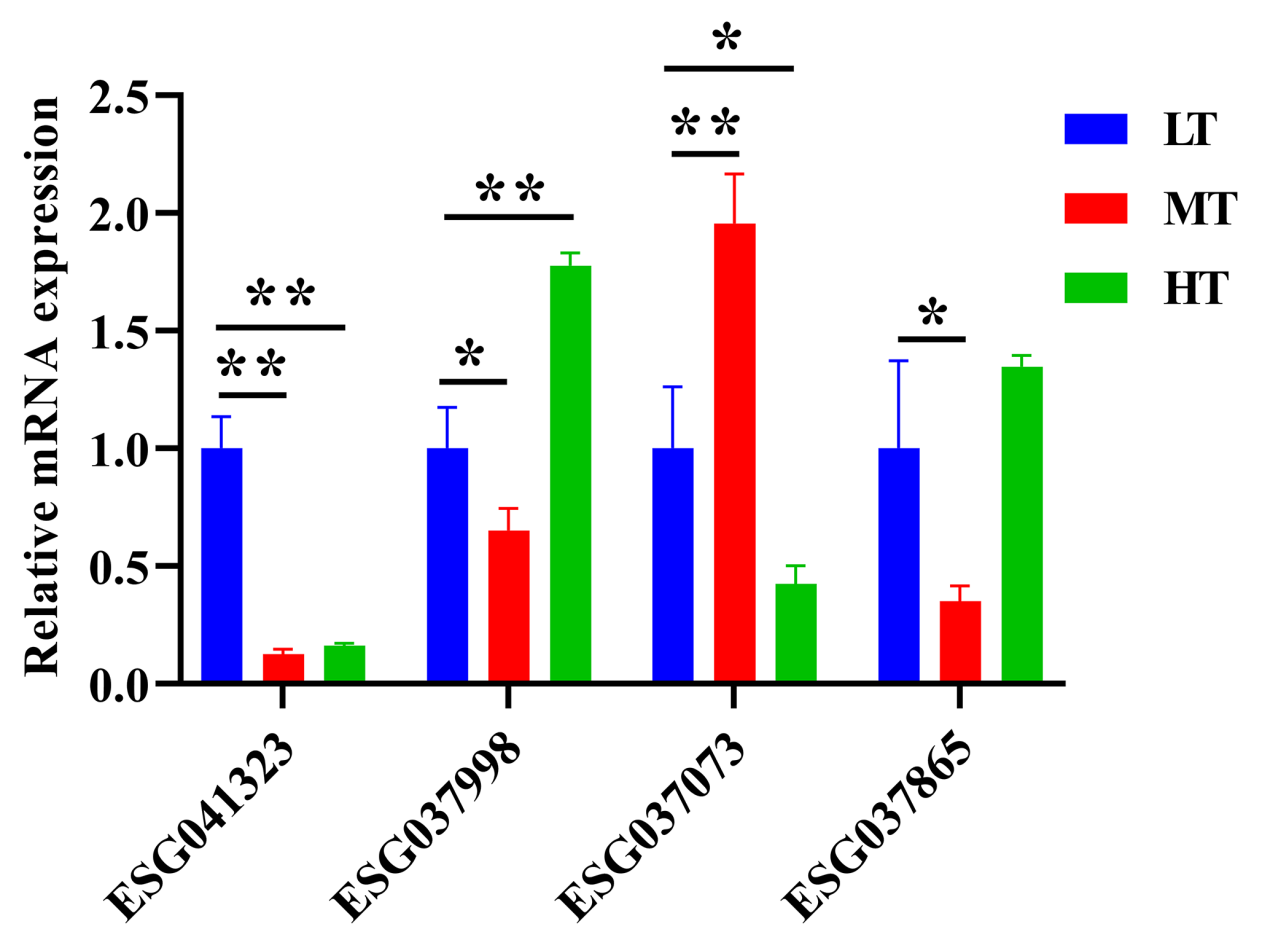
Validation of the RNA-seq profiles using quantitative reverse-transcription PCR (qRT-PCR). The relative expression of differentially expressed genes involved in the temperature adaptation-related pathways was detected. Y-axis represents the relative expression of each gene. Differences in the relative expression values for each gene were evaluated by *t*-test (n = 3). *p < 0.05, **p < 0.01

To understand the molecular mechanism underlying the response of *E. superba* to heat stress, we examined the expression of genes involved in the temperature adaptation-related pathways. Four DEGs involved in the temperature adaptation-related pathways were validated using qRT-PCR. In agreement with the results of RNA-seq, qRT-PCR findings confirmed that the expression of ESG041323, ESG037998, and ESG037865 was significantly down-regulated, while ESG037073 was markedly up-regulated in the MT group compared with the LT group (Fig. 4). In addition, in comparison with the LT group, the expression of ESG041323 and ESG037073 was markedly down-regulated, while ESG037998 expression was notably up-regulated in the HT group.

## Discussion

High-throughput transcriptome sequencing is an important tool for functional genomics research (Sudhagar et al. 2018). Using transcriptomic approaches, differences in gene expression between different tissues, developmental stages or under different stress conditions can be detected rapidly allowing for its wide application in immunity, stress, reproduction, and developmental biology research in aquaculture (Chandhini and Kumar 2019). However, little is known about the transcriptional changes that facilitate temperature adaptation in *E. superba*. In this study, we performed transcriptome sequencing on *E. superba* exposed to three different temperature conditions. In total, 1623, 142, and 842 DEGs were acquired in MT versus LT, HT versus LT, and HT versus MT, respectively. These DEGs were primarily associated with glucose metabolism, protein processing and cellular activities, and mainly participated in the Hippo signaling pathway, MAPK signaling pathway, and Toll−like receptor signaling pathway. This study provides rich resources for further research on *E. superba* and its growth under different temperature conditions.

Stress responses could compromise protein function by damaging cellular structures and thus deliver stress signals to the organisms (Imrie and Sadler 2012; Ron and Walter 2007). Therefore, organisms must respond to stress and maintain homeostasis. Studies have demonstrated that in response to stress, organisms undergo alterations in the structure and function of numerous enzymes and structural proteins. This leads to the synthesis of diverse stress response proteins, such as heat shock proteins, which are specifically designed to safeguard the organism from detrimental changes brought about by the stressors (Parsell and Lindquist 1993). In our study, GO analysis revealed that DEGs were primarily related to protein processing and cellular response to stress in *E. superba.* In agreement with our results, a recent study found that cellular activities and cellular processes in kuruma shrimp (*Marsupenaeus japonicus*) were also impacted by heat stress and *M. japonicus* produced adaptive responses to maintain physiological homeostasis through the transcriptional up-regulation of heat shock proteins and antioxidant enzymes (Zheng et al. 2019). Another study found that Arctic charr (*Salvelinus alpinus*) responded to increasing temperature by enhancing the expression of heat shock proteins and ubiquitin (Quinn et al. 2011). Therefore, we speculated that *E. superba* might avoid cellular damage caused by higher temperatures via protein processing and cellular activities.

Rising temperatures can induce various responses in shrimp, including changes in energy metabolism, heat stress, signaling and protein stabilization, which are common responses to environmental change (Hofmann and Todgham 2010; Zhou et al. 2010). Eukaryotes respond to heat stress by activating MAPK signaling pathways. MAPK signaling pathway is mainly composed of extracellular signal-regulated kinases, c-jun amino-terminal kinases and p38 MAPK. P38 MAPK subfamilies are primarily involved in inflammatory and environmental stress (UV irradiation, hyperosmolarity, heat shock response) responses (Winkler et al. 2002). Feidantsis et al. found that the p38 signaling pathway was involved in the regulation of heat shock protein Hsp70, protecting red blood cells under high temperature stress in gilt-head sea bream (*Sparus Aurata*) (Feidantsis et al. 2012). In addition, Anestis et al. suggested that the phosphorylation levels of p38 signaling pathway increase with increasing environmental temperatures in *Mytilus galloprovincialis* (Anestis et al. 2007). In this study, DEGs were shown to be involved in the MAPK signaling pathway. Therefore, the MAPK pathway might be involved in heat stress response in *E. superba*.

In conclusion, we performed transcriptome analysis of *E. superba* exposed to different temperatures and we obtained 1,623, 142, and 842 DEGs in MT versus LT, HT versus LT, and HT versus MT, respectively. These DEGs were primarily linked to the Hippo signaling pathway, MAPK signaling pathway, and Toll−like receptor signaling pathway. Altogether, the present study provides a suitable foundation for further research into the temperature adaptation mechanisms in *E. superba*.

## Data Availability

The datasets used and/or analyzed during the current study are available from the corresponding author upon reasonable request.
